# Severe Disseminated Infection with Emerging Lineage of Methicillin-Sensitive *Staphylococcus aureus*

**DOI:** 10.3201/eid2501.180684

**Published:** 2019-01

**Authors:** Paul Jewell, Luke Dixon, Aran Singanayagam, Rohma Ghani, Ernie Wong, Meg Coleman, Bruno Pichon, Angela Kearns, Georgina Russell, James Hatcher

**Affiliations:** Imperial College Healthcare NHS Trust, London, United Kingdom (P. Jewell, L. Dixon, A. Singanayagam, R. Ghani, E. Wong, M. Coleman, G. Russell, J. Hatcher);; Public Health England, London (B. Pichon, A. Kearns)

**Keywords:** Antimicrobial resistance, bacteria, epidural abscess, flucloxacillin, methicillin-resistant *Staphylococcus aureus*, methicillin-sensitive *Staphylococcus aureus*, MRSA, MRSA and other staphylococci, MSSA, psoas abscess, retropharyngeal abscess, Staphylococcus aureus, virulence factors

## Abstract

We report a case of severe disseminated infection in an immunocompetent man caused by an emerging lineage of methicillin-sensitive *Staphylococcus aureus* clonal complex 398. Genes encoding classic virulence factors were absent. The patient made a slow recovery after multiple surgical interventions and a protracted course of intravenous flucloxacillin.

Since being identified in 2002, methicillin-resistant *Staphylococcus aureus* (MRSA) clonal complex 398 (CC398) lineage, associated with livestock, has been a global public health concern (*1*). We know less about human infections with methicillin-sensitive *Staphylococcus aureus* (MSSA) CC398. Evidence suggests MRSA CC398 and MSSA CC398 are of distinct lineages (*2*); recently, MSSA CC398 has emerged as an invasive pathogen in humans without prior contact with animals (*3*). Here, we describe a case of severe disseminated MSSA CC398 infection in an immunocompetent man with no exposure to livestock. 

Shortly after arriving in the United Kingdom, a 60-year-old man from Colombia sought medical care after experiencing malaise, sore throat, and joint and muscular pain for 7 days. At hospital admission, he had signs consistent with sepsis and evidence of spreading soft tissue infection. Results of admission blood tests included leukocytes 49.9 × 10^9^/L (neutrophils 48.4), platelets 113 ×10^9^/L, C-reactive protein 437 mg/dL, creatine kinase 1,277 IU/L, lactate 4.5 mmol/L, and albumin 19 g/L. Laboratorians isolated MSSA from blood cultures taken at admission. Antimicrobial susceptibility testing using disc diffusion methodology (EUCAST version 8.0, http://eucast.org) showed susceptibility to cefoxitin, rifampin, ciprofloxacin, trimethoprim, and tetracycline, but resistance to penicillin, erythromycin, and clindamycin. Oxacillin MIC testing confirmed flucloxacillin sensitivity (MIC 0.38 mg/L).

Computed tomography and magnetic resonance imaging (MRI) images taken at admission indicated pyomyositis of the left subscapularis, inferior scalene, and intercostal muscles; a large retropharyngeal (Figure, panels A, B) and right psoas collection of pus, bibasal lung consolidation; and an epidural collection of pus extending from the lower thoracic to the lumbar spine and into the paraspinal soft tissues (Figure, panels C, D). HIV testing; hepatitis A, B, and C testing; myeloma screening; and a transthoracic echocardiogram all returned negative results. A transesophageal echocardiogram was not performed.

**Figure:**
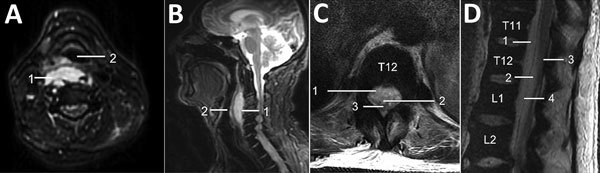
Magnetic resonance imaging of a 60-year-old immunocompetent man with methicillin-resistant *Staphylococcus aureus* clonal complex 398 infection. A, B) Axial (A) and sagittal (B) T2-weighted fat-suppressed sequences of the cervical spine demonstrate a retropharyngeal abscess (1) that moderately anteriorly displaces and mildly effaces the hypopharynx (2). C, D) Axial (C) and sagittal (D) T2-weighted MRI sequences of the thoracolumbar spine (T11–L2 vertebra levels labeled) demonstrate a large ventral, combined epidural (1) and subdural (2) spinal collection that displaces the conus medullaris (3) dorsally. Note the dura mater (4) on the sagittal sequence, which delineates the theca and separates the epidural and subdural spaces.

The patient was treated with intravenous flucloxacillin (2 g every 4 h) and rifampin (300 mg 2×/d) throughout his hospital admission. Initial surgical interventions consisted of incision and drainage of the retropharyngeal, right deltoid, and left subscapular collections. A second MRI, taken on day 13 of admission, showed discitis and osteomyelitis of the C3 and C4 vertebrae, return of the retropharyngeal collection, and enlargement of the psoas and epidural collections, with evidence of compression of the distal cord. No neurosurgical intervention was undertaken because the patient showed no focal neurologic signs. 

Despite images showing initial enlargement of collections, the patient experienced a slow clinical and biochemical recovery. MSSA was recovered from blood cultures until day 10, but tests were negative thereafter. A second computed tomography image, taken on day 30, showed a reduction in the size of the epidural and psoas collections. The patient returned to Colombia on medical grounds on day 46 to continue intravenous antibiotic therapy. 

Whole-genome sequencing showed the MSSA to be multilocus sequence type (ST) 4371 (a single-locus variant of ST398). Sequencing also found no genes encoding superantigens (9 enterotoxins and *tst*), exfoliatins, or Panton-Valentine leukocidin. Presence of the immune evasion cluster genes and the canonical single-nucleotide polymorphisms described by Stegger et al. (*4*) confirmed the isolate belonged to the human clade of CC398. Resistance genes *blaZ* and *erm(T)* were identified, correlating with the observed phenotype. 

This case of severe, widely disseminated infection in an immunocompetent man caused by a strain of MSSA without classic virulence factors is consistent with a growing body of evidence in support of MSSA CC398 as an emerging human pathogen. In cohorts of patients in France, CC398 MSSA has increased from being found in no cases in 1999 to 4.6% of cases in 2010 and 13.8% of cases in 2014 (*3*). Reports further indicate that nasal colonization with MSSA CC398 has increased in Europe (*5*). 

A study in New York, New York, USA, found MSSA CC398 infection to be associated with a largely Dominican neighborhood, in particular; Hispanic ethnicity was a clinical predictor (*6*). Another case of invasive MSSA CC398 in a patient from Colombia has also been described (*7*). 

Existing literature demonstrates evidence for MSSA CC398 as both a community- and hospital-associated pathogen (*3*). As in this case, MSSA CC398 has been shown to cause bloodstream infections (*8*), bone and joint infections (*5*,*9*) and skin and soft tissue infections (*3*,*7*). MSSA CC398 has also been associated with causing severe infection. Bouiller et al. found that 30-day all-cause mortality was higher for patients with CC398 MSSA bloodstream infection than for a control group with non-CC398 MSSA infection (*3*). Other cases of MSSA CC398 in the literature report similar resistance profiles and lack of virulence factors as we describe (*10*).

This case report aligns with existing evidence for MSSA CC398 as an emerging human pathogen. However, it is unusual in its degree of severity, with multiple extensive foci of infection, despite this strain lacking classic virulence factors. This lineage is of increasing global public health concern, and potential unidentified virulence factors and uncharacterized transmission patterns remain to be determined. 
